# Mapping the underlying drivers of resistome risk across diverse environments

**DOI:** 10.21203/rs.3.rs-7085902/v1

**Published:** 2025-10-23

**Authors:** Monjura Afrin Rumi, Loc Nguyen, Benjamin C. Davis, Connor L. Brown, Amy Pruden, Liqing Zhang

**Affiliations:** Virginia Tech; Virginia Tech; Environmental Protection Agency; Virginia Tech; Virginia Tech; Virginia Tech

**Keywords:** antibiotic resistance, one health, resistome risk, coverage, taxonomic diversity, regression

## Abstract

**Background:**

Understanding the drivers of antimicrobial resistance (AMR) across the One Health spectrum is crucial for controlling its spread. The MetaCompare framework, which assesses “resistome risk” based on antibiotic resistance gene (ARG) co-occurrence patterns on metagenomic contigs, has been expanded to distinguish between “ecological resistome risk” (ERR) and “human health resistome risk” (HHRR) scores across anthropogenic gradients. However, comprehensive surveys are still needed to untangle the biological (e.g., ARG relative abundance), ecological (e.g., taxonomic diversity), and technical (e.g., coverage) factors influencing these risk scores. Here, we analyzed 1,326 metagenomes from 12 key environments using the MetaCompare 2.0 pipeline to map global ERR and HHRR landscapes, identifying significant factors modulating risk scores through network analysis, machine learning, and multivariate regression models.

**Results:**

ERR and HHRR scores varied significantly across environments and were highly correlated (ρ = 0.73, p < 2e-16), indicating shared underlying drivers. Transient environments closely linked to human activity, such as wastewaters and the human gut, produced the highest ERR and HHRR scores, while stable environments like sediments, soils, and activated sludge yielded the lowest. These patterns corresponded directly with taxonomic diversity, where more diverse ecosystems exhibited lower risk scores, supporting the hypothesis that niche occupation may act as an ecological barrier to ARG invasion. In contrast, scores were positively correlated with *sul1* and crAssphage, further confirming that transient, low-diversity environments have higher resistome risks, although they did not fully account for risk variability across all environments. ARG relative abundance correlated with risk scores, but only in high-diversity, low-coverage environments due to poor assembly quality and an inability to resolve ARG flanking regions. The ARGs contributing to ERR and HHRR scores were largely aligned with existing ARG risk ranking frameworks.

**Conclusions:**

This study demonstrated how the MetaCompare 2.0 pipeline can effectively disentangle complex relationships between ARG abundance, composition, and environmental context. Although robust across diverse environments, the framework’s ability to detect ARGs and their co-occurrences may be limited in high-diversity, low-coverage samples, such as soils and sediments. Finally, we provide a series of recommendations for appropriate use cases for MetaCompare 2.0.

## Introduction

Antimicrobial resistance (AMR) refers to the ability of microorganisms to survive exposure to antimicrobial agents, such as antibiotics. AMR poses a critical threat to global public health, making infections harder to treat, increasing healthcare costs, and elevating morbidity and mortality rates. In 2021, AMR was estimated to be responsible for approximately 4.71 million deaths, with models predicting up to 8.22 million deaths globally by 2050 [1]. Bacterial resistance to antibiotics is encoded by antibiotic resistance genes (ARGs), which can spread among bacterial populations, including both pathogenic and non-pathogenic species, through horizontal gene transfer (HGT). The role of the environment in the evolution and dissemination of AMR has become a focal point in advancing science-based policy to stem the spread of AMR [2–5]. Natural environments, such as soil, have been identified as reservoirs for ARGs that encode resistance to antibiotics used in human medicine, making their way into human pathogens via HGT [2–6]. Anthropogenic inputs to soil and aquatic environments, such as wastewater, manure, and industrial discharges, can introduce resistant bacteria, pathogens, and selective agents at concentrations that may promote HGT and the proliferation of ARGs [7]. HGT is of particular concern as it facilitates the rapid evolution of resistance in bacterial populations by allowing the acquisition of ARGs from diverse sources. A variety of mobile genetic elements (MGEs), including plasmids, integrative conjugative elements, transposons, insertion sequences, and integrons, play pivotal roles in the capture and spread of ARGs [8–9]. A metric to assess the potential for ARGs to transfer horizontally into pathogens across the One Health spectrum, spanning human, animal, plant, and environmental health, would provide valuable insight towards informing effective strategies to combat AMR as a global threat [2–6, 8–9].

To estimate the potential for ARGs to mobilize into pathogens, we developed the MetaCompare framework based on the concept of “resistome risk” [10], which is defined as the relative potential that ARGs may mobilize into pathogens within a given environment based on the co-occurrence patterns of ARGs, MGEs, and pathogens on metagenomic contigs [11]. Building on this approach, MetaCompare 2.0 (MC2) was developed to further refine resistome risk scores by distinguishing between “ecological resistome risk (ERR)” and “human health resistome risk (HHRR)” [12]. HHRR provides a metric representing the degree to which gene markers for ESKAPEE pathogens (i.e., *Enterococcus faecium, Staphylococcus aureus, Klebsiella pneumoniae, Acinetobacter baumannii, Pseudomonas aeruginosa, Enterobacter* spp. and *Escherichia coli*) co-occur with MGEs bearing Rank I ARGs deemed to be of acute concern for human health [13], while ERR more broadly assesses the ARG mobility potential by including a comprehensive list of known pathogens. Together, HHRR and ERR show promise for comparing environments and identifying hotspots for antibiotic resistance dissemination, guiding possible mitigation strategies.

The MC2 framework disentangles the non-direct proportionality between abundance and risk by considering the composition and context of detected ARGs [14]. Correspondingly, it has proven effective in distinguishing samples from various environments sub-categorized as high and low pollution/anthropogenic impact, offering a practical tool for identifying and prioritizing ARG hotspots for targeted mitigation. MC2 was successfully benchmarked to several technical factors that could confound risk scores, such as sequencing depth, coverage, and assembly algorithms, but it is possible that other underlying biological and ecological factors could still influence computed scores in undefined ways. A fundamental understanding of the biological drivers of resistome risk scores is still lacking, as is a comprehensive mapping of the global resistome risk landscape across diverse environments.

Recently, it was hypothesized that overall microbiome stability can prevent the invasion of exogenous ARG-carrying bacteria, curbing the mobility of ARGs [15–16]. As shown by Klumper et al. [15], environmental microbiome diversity can act as a natural defense against ARG invasion when comparing the dynamics of native bacterial diversity and ARG abundance in polluted versus unpolluted soil and river environments. In ecosystems with high taxonomic diversity, resource niches are more efficiently occupied, which limits the ability of allochthonous ARG-carrying bacteria to establish residence [16]. However, anthropogenic stressors can disrupt community assemblages and alter the effective niche occupation of the resident microbes [17–18]. In such disturbed systems, invaders face less competition for resources, increasing their potential for long-term colonization and HGT of their ARGs, thus resulting in the spread and persistence of antibiotic-resistant bacteria (ARB) [19–20].

**Figure 1:**
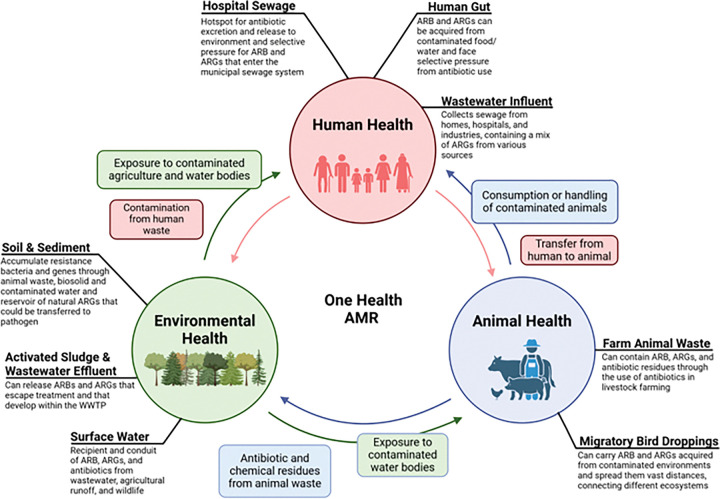
Selection of environments included in this study and their relationship to AMR evolution and dissemination concerns across the One Health spectrum.

In the present study, we further hypothesized that environments characterized by high microbial diversity and stability would exhibit lower resistome risk due to their resilience to the invasion of exogenous ARB. In contrast, environments subject to anthropogenic stressors, such as wastewater and agricultural runoff, would be expected to exhibit higher resistome risk, creating conditions that favor the invasion and accumulation of ARGs. We utilized publicly-available metagenomic datasets comprising 1,326 samples from 12 distinct environments (wastewater influent, activated sludge, wastewater effluent, hospital sewage, surface water, soil, sediment, cow manure, pig feces, chicken feces, migratory bird droppings, and human gut) that capture various dimensions of concern for AMR dissemination across the One Health spectrum (Fig. 1). This dataset was analyzed using a combination of network analysis, machine learning and multivariate regression models to: i) characterize the global resistome risk landscape, ii) determine key biological/ecological drivers of resistome risk, and iii) identify key ARGs and MGEs that most influence risk scores. We measured abundance of crAssphage, which has recently emerged as a reliable indicator of human fecal contamination [21–22], and *sul*1, which has been shown to be strongly correlated with anthropogenic gradients [23–24], as a means to quantify the degree of anthropogenic impact. This study provides a deeper understanding of underlying factors driving the ecology and evolution of AMR in various environments and further evaluation of resistome risk as a useful metric for prioritizing mitigation efforts.

## Methods

### Data Collection

**Figure 2:**
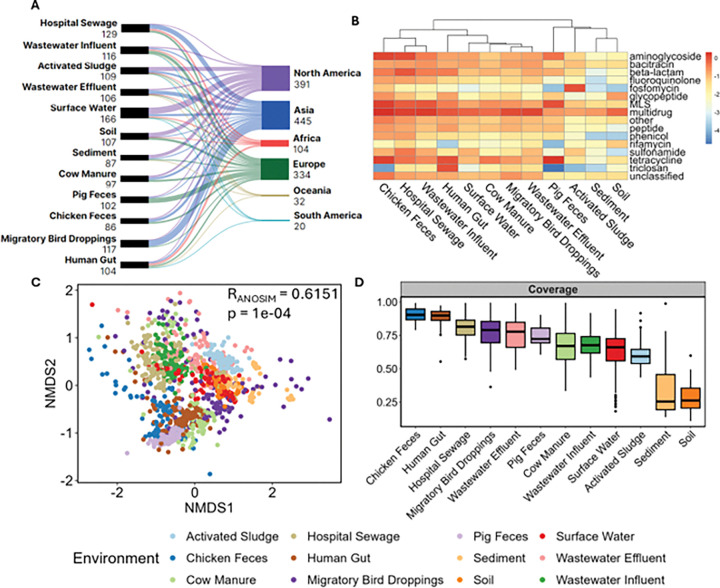
(A) Overview of the metagenomic datasets obtained from the NCBI SRA database for this study, including the number of samples corresponding to each environment category and their global distribution. (B) The log_10_ of the average ARG relative abundance (ARG copies/cell) observed across the environments, with the corresponding antibiotic resistance class indicated. (C) NMDS of Bray-Curtis dissimilarity based on the overall ARG relative abundance across all environments. (D) Boxplots of coverage value across all samples ranked by the highest average to lowest average.

We searched the SRA database in NCBI using the search terms listed in Supplementary Table S1 to identify publicly-available metagenomic sequencing data obtained from 12 distinct environments across the globe that are representative of various environmental dimensions of One Health AMR concerns (Fig. 1). The identified samples were further screened to ensure they were sequenced on an Illumina platform with 100–150bp read length and between 3–20 GB per sample. Paired-end raw sequence data was downloaded from the resulting 1,326 sample entries that met these criteria and were broadly binned after literature review as representative of the 12 environments of interest. The data collection process is shown in Supplementary Figure S1. The number of samples collected for each environment and their continental location is shown in Fig. 2A. Detailed descriptions including the location, SRA accession and BioProject ID for the samples are summarized in Supplementary Table S2.

### Bioinformatic Analysis

Fastp was applied to filter out low-quality reads by enabling parameters to remove adapter information and trim polyX and polyG reads [25]. Coverage of the samples was estimated by applying Nonpareil 3.3 with option ‘-T kmer’ [26]. Forward and backward reads were merged with VSEARCH [27], then aligned against DeepARG-DB [28] using DIAMOND [29] (80% identity, min 25aa), and normalized to ARGs/cell (*rpoB*) [30] using DIAMOND [29] (40% identity) to obtain the abundance of ARGs in the samples. MGEs were identified by using DIAMOND [29] against the MobileOG-db [31] and their abundance was calculated using an analogous procedure as was carried out for ARGs. Short reads were also taxonomically annotated using Kraken2 [32] against the Standard-16 database and the relative abundances at the genus level were then estimated using Bracken [33]. Shannon diversity using the short read taxonomic annotations was calculated using KrakenTools [34]. CrAssphage and *sul1* gene abundance was measured using the methods from Karkman et al. [21]. Short paired-end reads were mapped against the CrAssphage (NC_024711.1) and *sul1* gene reference sequence using bowtie2 [35] and genome coverage for the mapped reads was calculated using SAMtools [36]. Short reads were assembled through MEGAHIT [37] with default parameters and subsequently processed via the MC2 [12] pipeline to calculate ERR and HHRR scores.

### Statistical analysis

Alpha diversity of ARGs was calculated using the Shannon index from the vegan [38] package. The Levins’ niche breadth index was calculated using the MicroNiche [39] package to determine whether an ARG is considered a generalist or specialist across all environments. To assess factors influencing resistome risk, two linear regression models were employed. The first model assessed the impact of various environmental and genomic parameters on resistome risk scores. Here, resistome risk score was the dependent variable, while sequencing coverage, ARG abundance, MGE abundance, ARG diversity, MGE diversity, taxonomic diversity, number of contigs, and N50 contig length served as independent variables. Statistical significance was set at a p-value < 0.05, adjusted using the Benjamini-Hochberg method [40]. The second regression model explored the relationship between resistome risk scores and specific risk-determining parameters defined within the MC2 pipeline: Q_ARG_, Q_ARG MGE_, Q_ARG,PAT_, and Q_ARG,MGE,PAT_. To ensure comparability across these variably-scaled independent variables, standardized coefficients were computed for all analyses.

Normality of all datasets was assessed using the Shapiro-Wilk test. As all datasets were found to be non-normally distributed, non-parametric statistical methods were subsequently applied. Spearman’s correlation was conducted to evaluate the relationship between ERR and HHRR, as well as the association between risk scores and the abundance of crAssphage and *sul1*. The ANOSIM test was conducted to analyze pathogens co-occurring with ARGs and resistome profile across environments.

### Regression Analysis

We conducted a regression analysis for each environment to identify the ARGs that contributed significantly to the resistome risk scores. ARGs detected in all samples representing a given environment were selected as feature set and sample risk score was chosen as the dependent variable. To quantify the contribution of individual ARGs, we calculated a modified risk score for each ARG. This involved applying a modified version of the MC2 approach: for each ARG, it was treated as the sole ARG present in the sample, and all risk calculation parameters were determined under this assumption. The final individual ARG risk score was then calculated following the original MC2 methodology. To identify the ARGs that contributed the most to the resistome risk score, we applied a feature selection method using Random Forest regression [41]. This process was separately conducted for both ERR and HHRR. The regression analysis was performed using the scikit-learn [42] library. Using the regression model, we also determined the co-occurring ARG-MGEs that significantly contribute to the risk scores.

### Network Analysis

Building on the MC2 analysis of each sample, we additionally constructed ARG-MGE co-occurrence networks for each environment using all the samples representing a given environment. In each sample, an ARG was defined as mobile if it co-occurred with an MGE and represented in the network using an edge whose one node is the ARG and the other node is the MGE. For each edge e, an edge weight (e_w_) was set based on the normalized frequency in the samples. If the edge (e) occurred in m number of samples and N is the number of total samples analyzed, edge weight was calculated as e_w_=m/N. To focus on robust associations, edges with an edge weight less than 5% (i.e., observed in fewer than 5% of samples) were filtered out (Supplementary Figure S2). In a similar fashion, we also constructed ARG-MGE-Pathogen networks for each environment. For these three-node networks, the criterion was that mobile ARGs must be carried by pathogens. The networks were subsequently explored to determine frequently occurring mobile ARGs and pathogens carrying such ARGs in different environments.

All plots in this paper were visualized using ggplot2 [43], pheatmap [44] and networkD3 [45] in R, and NetworkX [46] in Python.

## Results

### Assessing ARG Abundance, ARG Diversity and Metagenomic Coverage across Different Environments

We systematically examined a set of 1,326 samples from 74 countries across 12 environment types to assess ARG abundance, ARG diversity, taxonomic diversity, and metagenomic coverage and their relationship with resistome risk (Fig. 2A). To evaluate the robustness of the dataset for making cross-environment ecological assessments, we first estimated metagenomic coverage. Although samples were globally sourced, coverage was largely dependent upon environment type and strongly negatively correlated with taxonomic diversity (R=−0.737, p < 2e-16). Among the twelve environments analyzed, coverage ranged from an average of 91,87, and 81% in chicken feces, human gut, and hospital sewage samples, to 34 and 28% in sediments and soils respectively (Fig. 2D, Table S4). We found that 386/1,326 (29.1%) samples were under sequenced with coverages less than 60%, over half of which (62.1%) belonged to sediment, soil, and activated sludge samples.

To evaluate patterns of antibiotics resistance across all environments, we profiled the ARG composition and abundance of our samples. Nine drug classes (Fusaric acid, Tetracenomycin C, Fusidic acid, Acridine dye, Aminocoumarin, Mupirocin, Bicyclomycin, Pleuromutilin, polyamine:peptide) were found in very low abundance (average < 0.01 ARG copies/cell) in all environments (Supplementary Table S3). In contrast, ARGs that confer multidrug resistance were detected in high abundance (average > 0.05 ARG copies/cell) in every environment (Supplementary Table S3, Fig. 2B). Drug classes with extremely high abundance (>1 ARG copies/cell) were observed for macrolide-lincosamide-streptogramin (MLS) and tetracycline in chicken and pig feces, aminoglycoside in chicken feces and hospital sewage, and multidrug resistance in chicken feces, wastewater effluent, hospital sewage, wastewater influent and migratory bird droppings (Supplementary Table S3, Fig. 2B). On average, hospital sewage, chicken feces, and wastewater influent samples displayed the highest relative abundance of ARGs, while soils, activated sludge, and sediment samples had the lowest (Supplementary Table S3, Fig. 2B). The resistome composition was significantly different across environments, as revealed by non-metric multidimensional scaling (NMDS) and analysis of similarities (ANOSIM; R_ANOSIM_ =0.6151, p = 1e-04) based on Bray-Curtis index (Fig. 2C).

### Global Resistome Risk Landscape

**Figure 3:**
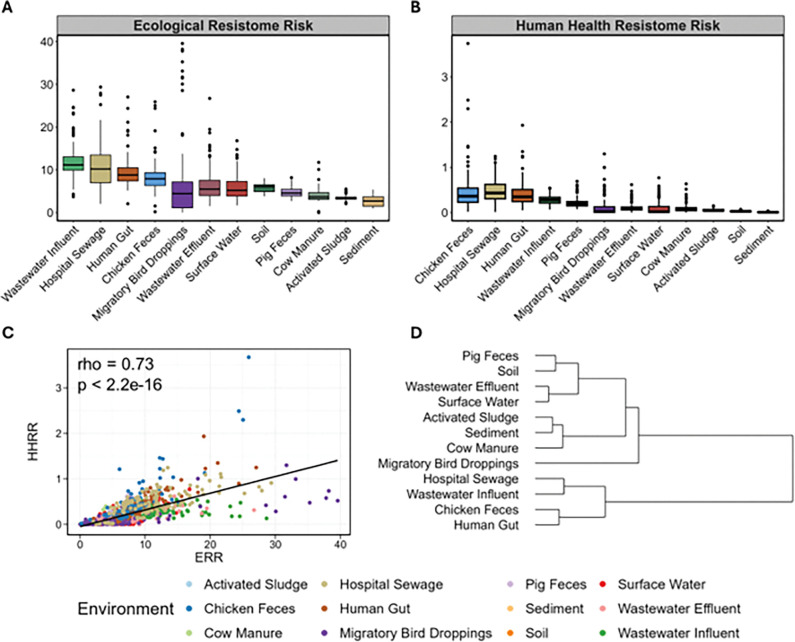
Distribution of A) ERR and B) HHRR across all environments in the dataset ranked by highest to lowest average. C) Spearman correlation between ERR and HHRR scores across all environments. (D) Dendrogram of environment groupings based on the Wasserstein distance of ERR and HHRR scores.

Similar to resistome composition, both ERR and HHRR scores were significantly different across the 12 environments (Kruskal-Wallis, p-value < 2.2e-16). Environments directly related to humans; wastewater influent (11.8), hospital sewage (10.9), and human gut (9.6) samples exhibited the highest average ERR scores, while activated sludge basins (3.5) and sediments (2.7) yielded the lowest (Supplementary Table S4, Fig. 3A & 3B). For HHRR, scores were lower than ERR scores and ranged from an average of 0.52 in chicken feces to 0.01 in sediments. The highest HHRR score (3.7) was observed in feces from a broiler chicken in China. Using pairwise Wasserstein distances of ERR and HHRR scores distributions, we observed four distinct groupings: (1) pig feces, soil, wastewater effluent, and surface water; (2) activated sludge, sediment, and cow manure; (3) migratory bird droppings; and (4) hospital sewage, wastewater influent, chicken feces, and human gut (Fig. 3D).

ERR and HHRR were found to be strongly positively correlated across samples, suggesting a set of shared underlying stressors across environments (R = 0.73, p < 2.2e-16) (Fig. 3C). We observed that chicken feces, human gut, hospital sewage, and pig feces deviated towards the HHRR side of the linear regression slope indicating a proportional enrichment in ESKAPEE pathogens and mobile Rank 1 ARGs (Fig. 3C). Reciprocally, we observed that activated sludge, cow manure, migratory bird droppings, sediment, soil, surface water, wastewater effluent and wastewater influent were proportionally higher in ERR scores, indicating a high density of mobile ARGs carried by more diverse bacterial pathogens (Fig. 3C).

### Technical and Biological Factors Underlying Risk Scores

To explore the underlying drivers of resistome risk across environments, we analyzed the impact of both technical (e.g., coverage, contig N50s, and number of contigs) and biological factors (e.g., ARG and MGE abundance (gene copies/cell), ARG, MGE, and taxonomic diversity) using a multi-linear regression (See the raw data in Supplementary Table S5). As an integration of both technical (sequencing depth) and biological (diversity) biases, metagenomic coverage serves as an indication of the robustness of the MC2 pipeline. Here, we observed that while coverage was generally positively correlated with ERR and HHRR, only a few environments showed significant positive correlations between both scores: sediment (R^2^ = 0.88, p = 1.35e-03), activated sludge (R^2^ = 0.59, p = 4.30e-02), human gut (report R^2^ = 0.57, p = 7.41e-04), and pig feces (R^2^ = 0.91, p = 6.68e-18) for ERR, and wastewater influent (R^2^ = 0.69, p = 1.10e-04), pig feces (R^2^ = 0.82, p = 2.70e-06), and surface water (R^2^ = 0.74, p = 1.40e-02) for HHRR (Supplementary Figure S3D & S4D). Further, we observed that both ERR and HHRR scores were significantly correlated with total ARG abundance, but only in a subset of environments. Specifically, sediment, soil, and activated sludge ERR and HHRR scores were positively correlated with ARG abundance, whereas wastewater effluent, surface waters, and chicken feces showed largely insignificant or negative associations (Supplementary Figure S3A & S4A). Although there may be an increase in the “density” of ARGs on a per cell basis, they are not necessarily proportionally associated with MGEs or partitioned into human pathogens. Linear regression analysis on Q_ARG_, Q_ARG,MGE_ and Q_ARG,MGE,PAT_ indicated a correlation coefficient ~ 1.0 for Q_ARG_ with ERR in soil and sediment, whereas this correlation was very low for Q_ARG,MGE_ and Q_ARG,MGE,PAT_ (Supplementary Figure S5).

**Figure 4:**
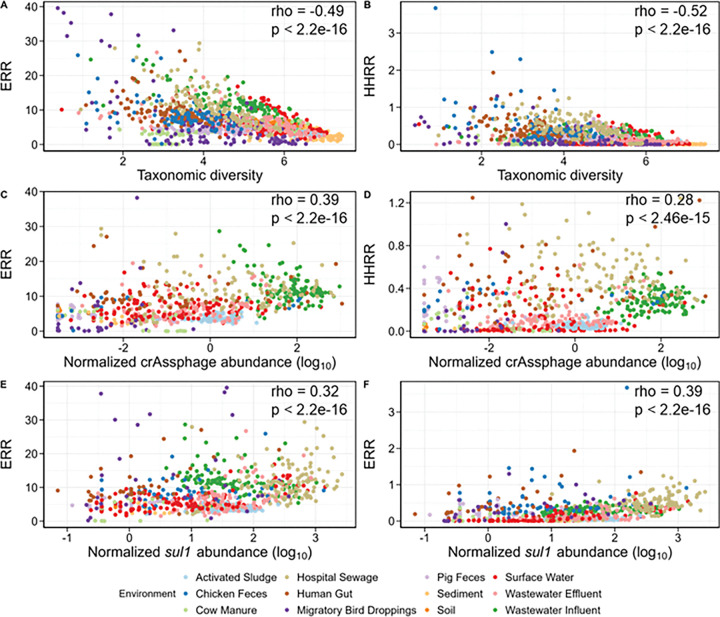
Spearman’s correlation of Shannon taxonomic diversity to (A) ERR and (B) HHRR. Spearman’s correlation of log-transformed log_10_() values for crAssphage (C-D) and *sul*1 (E-F) abundances to ERR and HHRR. Samples with zero mapped reads to the crAssphage or s*ul1* references were excluded prior to plotting (see Supplementary Table S7 for raw abundance values).

ARG diversity was found to be significantly positively correlated with both ERR and HHRR in wastewater influent, the human gut, surface water, and wastewater effluent (Supplementary Figure S3B & S4B). The number of contigs was found to be negatively correlated to both HHRR and ERR (Supplementary Figure S3G & S4G). In general, we observed moderately strong negative correlations between taxonomic diversity and ERR (R = −.049, p < 2e-16) and HHRR (R= −0.52, p < 2e-16) scores (Fig. 4A & 4B). Specifically, sediment, activated sludge, and soil samples had the highest taxonomic diversity, but displayed the lowest ERR and HHRR scores. Reciprocally, wastewater influent, hospital sewage, human gut, and chicken feces samples produced the highest resistome risk scores, but tended to be much less taxonomically diverse. Taxonomic diversity showed a significant negative correlation with ERR in seven environments, whereas there was minimal impact of taxonomic diversity on HHRR across most environments (Supplementary Figure S3C & S4C).

We quantified the abundance of crAssphage and the *sul1* gene and assessed their correlations with ERR and HHRR to evaluate the influence of anthropogenic impact on resistome risk scores. For both ERR and HHRR, a significant, but weak, positive correlation was observed with crAssphage and *sul1* (Fig. 4C, 4D, 4E & 4F). As expected, crAssphage levels were highest in wastewater influent and hospital sewage, which also had the highest ERR and HHRR scores (Supplementary Figure S6A). In contrast, soil and sediment samples contained the lowest crAssphage abundance and the lowest HHRR scores (Supplementary Figure S6A). Two notable exceptions were identified to this pattern: chicken feces and activated sludge. Chicken feces ranked in the middle for average crAssphage and *sul1* abundance but exhibited relatively high resistome risk scores. Activated sludge had high crAssphage and *sul1* abundance, but were characterized by relatively low resistome risk scores (Supplementary Figure S6A & S6B). The number of samples removed due to zero reads mapped to the crAssphage genome were: 65/86 chicken feces, 69/97 cow manure, 2/129 hospital sewage, 40/104 human gut, 90/117 migratory bird droppings, 64/102 pig feces, 74/87 sediment, 103/107 soil, 36/166 surface water, and 13/106 wastewater effluent (Supplementary Table S6). For *sul1*, samples removed were: 32/86 chicken feces, 80/97 cow manure, 67/104 human gut, 84/117 migratory bird droppings, 53/102 pig feces, 70/87 sediment, 103/107 soil, 23/166 surface water, and 5/106 wastewater effluent (Supplementary Table S6).

### ARGs that Most Impact Resistome Risk

**Figure 5:**
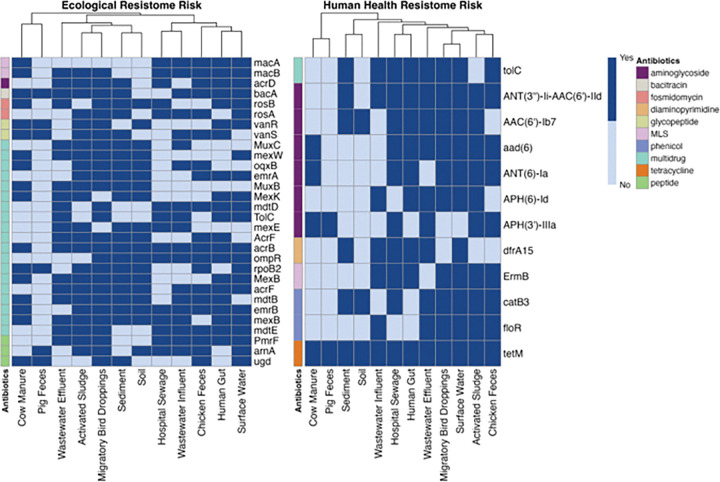
ARGs that substantially contributed (yes or no) to A) ERR and B) HHRR in ≥ 6 environments, as determined by Random Forest regression analysis (see the full list in Supplementary Tables S8 and S9).

To identify the key ARGs contributing to the resistome risk scores, we ran a separate random forest regression model for each environment. Individual resistome risk scores calculated for each available ARG in an environment were used as input data. To ensure robust predictions, only models with an R^2^ > 0.85 were considered. Feature selection within these accepted models identified 404 ARGs contributing substantially to ERR scores (Supplementary Table S8). Further analysis revealed that 105 of those 380 genes (27.6%) conferred resistance to beta-lactam, while 99(26.1%) were associated with multidrug resistance. A cross-check with the WHO list of medically-important antimicrobials showed that 256 genes (66.4%) overlapped with the WHO list. Similarly, using data from the HHRR calculation pipeline, 182 unique genes were identified that substantially contributed to the HHRR score (Supplementary Table S9). Among these 182 genes, 102(56.04%) conferred resistance to beta-lactams. Notably, 171(94.0%) of these genes conferred resistance to medically-important antimicrobials from the WHO list [47] (See citation for details). We compared our list of ARGs to those incorporated in the HHRR score as originally proposed as Rank I ARGs by Zhang et al. [13], who employed a ranking system based on three criteria: human-associated enrichment, gene mobility, and host pathogenicity. A total of 220 ARGs (54.5%) and 140 ARGs (76.9%) contributing to ERR and HHRR, respectively, were included in the ranking proposed by Zhang et al. [13] (Supplementary Table S8 & S9). Among the ARGs that substantially contributed to the ERR, *acrB* (multidrug) was the only substantial contributor in all environments except pig feces, followed by *mexB* (multidrug), *emrB* (fluoroquinolone) and *bacA* (bacitracin) in 10/12 environments (Fig. 5A). Based on the Levins’ niche breadth index, *acrB* and *bacA* are considered moderate generalist ARGs, with niche breadth values of 0.65 and 0.58, respectively. In contrast, *mexB* is classified as a moderate specialist (0.37), while *emrB* does not fall into either classification (0.53)(Supplementary Table S10). For ARGs substantially contributing to HHRR, *tetM* (tetracycline) was the only gene that substantially contributed to HHRR in all environments, followed by *ANT(3”)-Ii-AAC(6’)-IId* (aminoglycoside) in 9/12 environments (Fig. 5B). From the Levins’ niche breadth index, *tetM* is considered a moderate specialist ARG (0.37), while *ANT(3”)-Ii-AAC(6’)-IId* is considered an extreme specialist (0.09)(Supplementary Table S10).

### Co-occurrence of ARG and MGEs Driving Resistome Risk

**Figure 6:**
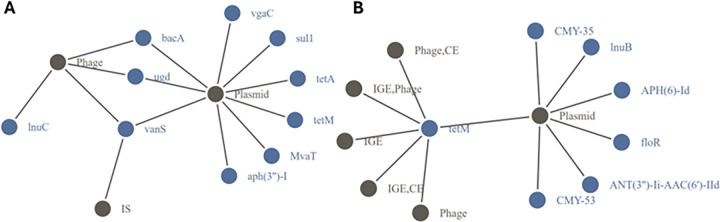
(A) ARG-MGE combinations that substantially contribute to ERR that were detected in ≥ 7 environments. (B) ARG-MGE combinations that substantially contribute to HHRR that were detected in ≥ 6 environments. Blue nodes represent ARGs, and brown nodes represent MGEs.

The network analysis identified specific co-occurrences of ARGs and MGEs, as well as the prevalence of common ARG-MGE combinations within and across different environments. ARG-MGE combinations from this network were used as input into the Random Forest [41] regression model to identify combinations that substantially contribute to the risk scores. For ARG-MGE combinations that substantially contributed to the ERR, we identified 664 unique combinations (Supplementary Table S11). The most shared ARG-MGE combinations were *bacA* (bacitracin) with a plasmid or phage and *vanS* (glycopeptide) with a phage. These combinations were found in all environments, except soil and sediment (Supplementary Table S11 & Fig. 6A). In this network, genes appearing in 22.3% of unique edges conferred resistance to multidrug, genes in 17.8% were associated with beta-lactams, and 15.1% conferred resistance to MLS antibiotics. For MGEs, plasmids were observed in 34.9% of unique edges, phages in 18.4% of cases, and 13.0% were integrative elements (IGEs). Similarly, we identified 282 unique ARG-MGE combinations that substantially contributed to the HHRR (Supplementary Table S12). The most frequent shared combination was *tetM* (tetracycline) with either an IGE, plasmid, or phage, found in all environments except activated sludge, soil and sediment (Supplementary Table S12 & Fig. 6B). In this network, 18.1% of all combinations conferred resistance to aminoglycoside, while 17.0% conferred resistance to cephalosporin, monobactam, penam and penem simultaneously and 9.6% to cephamycin. In the case of MGEs, 43.3% of all combinations were plasmids, 19.5% were IGEs, and 9.2% for phages. When examining medically important antibiotics as categorized in the World Health Organization (WHO) report “WHO List of Medically Important Antimicrobials” [47], we identified ARGs associated with 464 combinations (69.9%) among those ARG-MGE combinations substantially contributing to the ERR and 262 combinations (92.9%) substantially contributing to the HHRR (Supplementary Table S11 & S12).

### Properties of Pathogen Groups Co-occurring with ARGs

We assessed the abundances of pathogen-like contigs containing ARGs and used ANOSIM R statistics to evaluate similarities and differences in pathogenicity across various environments. Pathogens detected in human and animal feces, excluding migratory bird droppings, were found to be highly dissimilar from those found in activated sludge, soil, and sediment, as well as dissimilar from pathogens in other environments (Supplementary Figure S7). In contrast, surface water, wastewater effluent, and migratory birds harbored a broad range of pathogens, resulting in slightly similar to slightly dissimilar pathogenicity compared to other environments. From the ARG–MGE–pathogen co-occurrence network, Escherichia emerged as the most frequently detected pathogen co-occurring with an ARG and MGE, present in 34.5% of cases, followed by Acinetobacter (15.0%) and Prevotella (6.5%) (Supplementary Table S13). All three pathogens were identified in more than four environments.

## Discussion

The MC2 framework provides comparable metrics that enable users to evaluate the relative resistome risk within and across different environments. While the original MC2 study benchmarked technical factors that could confound risk scores [12], comprehensive analyses of both biological and technical factors influencing these scores are needed. In this study, we performed a comprehensive analysis to quantify the ERR and HHRR scores and evaluated the underlying technical, biological, and ecological factors that could influence these scores. To achieve this, we examined 1,326 metagenomic samples from 12 distinct and globally-distributed environments with varying taxonomic diversity.

Given that our dataset was compiled from multiple studies, we examined metagenomic coverage to assess the extent to which MC2 can provide robust metrics when challenged by deficiencies in the sequencing data. Coverage is a key defining feature of sequencing quality, as a low sequencing depth can affect assembly quality and potentially limit the biological representativeness of calculated risk scores. We observed that coverage did not generally vary among individual studies, but was mainly associated with sample type. Notably, soil and sediment samples produced the lowest coverage (averaging around ~ 30%), with the next lowest being activated sludge samples (averaging around ~ 60%), which is consistent with inherently high microbial diversity of these environments (Fig. 2C). ERR in these high-diversity, low coverage environments were dominated by Q_ARG_ (regression coefficient ~ 1), while near-zero coefficients were produced for Q_ARG–MGE_ and Q_ARG–MGE–PAT_, indicating that the risk score in these samples was primarily driven by the presence of ARGs alone rather than their co-occurrences (Supplementary Figure S5). This is potentially an artefact of poor assembly quality due to insufficient sequencing depth, limiting the detection co-occurrences with MGEs and pathogens. Additionally, we found that the number of contigs was generally negatively correlated with risk scores for the majority of environments (Supplementary Figure S3G & S4G). This may be due to a large proportion of contigs being too short to be annotated for ARGs and their flanking regions. As a result, the increased number of small, unannotated contigs inflates the denominator of the MC2 calculation pipeline, lowering the overall risk scores.

Despite the above limitations, using a multiple linear regression approach, we found that coverage had minimal impact on ERR and HHRR in low coverage environments (Supplementary Figure S3D & S4D). Although there was a significant positive correlation between coverage and ERR in sediment samples and a significant negative correlation between coverage and HHRR in activated sludge samples, the magnitude of the regression coefficients was low. Based on our observations, the risk scores generated by MC2 for complex samples remained stable even at low coverages. However, it remains difficult to fully disentangle the biological patterns from technical limitations. Specifically, limited detection of Q_ARG–MGE_ and Q_ARG–MGE–PAT_ in low coverage, high-diverse environments may reflect true biological scarcity or possibly an underestimation caused by fragmented assemblies. Therefore, caution is warranted when interpreting risk scores in samples with poor assembly quality.

Consistent with recent findings reported by Klumper et al. [15], we found that ARG abundance negatively correlated with taxonomic diversity across our dataset (Supplementary Figure S8A). We hypothesized that ARG invasion would be modulated by taxonomic diversity and therefore be a key ecological driver of resistome risk. Specifically, environments with high microbial diversity may offer more efficient niche occupation and ecological resistance to invasion, thus reducing the likelihood of exogenous ARG proliferation and lowering resistome risk. Our examination of publicly-available metagenomes support this hypothesis, as we observed that environments with high taxonomic diversity generally were characterized by lower resistome risk scores compared to less diverse environments (Supplementary Table S4). In examining how taxonomic diversity is associated with ERR and HHRR, we found that taxonomic diversity was negatively correlated with ERR in the majority of environments, but had minimal or non-significant positive correlations with HHRR (Supplementary Figure S3C & S4C). We suspect this discrepancy may be because HHRR calculations focus on Rank I ARGs as a subset of acute concern to human health, as well as those associated with the ESKAPEE pathogens. The presence of transient, fecal-associated organisms could potentially increase the HHRR by contributing to the detection of ARGs and MGEs linked to human infections. However, corresponding fecal-associated pathogens, which are captured in the ERR calculations, may not accurately represent the broader taxonomic diversity present across the environments.

We observed that transient environments, such as sewage, the human gut, and fecal samples, produced higher HHRR scores compared to stable environments, like sediment and soil (Fig. 3B). This phenomenon has been previously observed, where arable soils amended with sewage sludge were resilient to AMR enrichment over both short- and long-term scenarios [48]. However, to determine whether an environment’s resistome risk score is driven by the presence of ecologically integrated AMR determinants, or if it merely reflects the transient detection of ARB incidentally present in the sample, we quantified the abundance of crAssphage and *sul1* as indicators of exogenous anthropogenic inputs. As expected, there was a moderate, but significant positive correlation between both ERR and HHRR and the abundance of these anthropogenic markers (Fig. 4C–4F). The four environments with the highest levels of crAssphage and *sul1* were wastewater influent, wastewater effluent, hospital sewage, and activated sludge (Supplementary Figure S6), supporting the idea that anthropogenic inputs contribute to increased resistome risk. However, the relatively low resistome risk scores observed among the activated sludge samples may reflect their diverse and functionally stable microbial community acting as an ecological barrier to allochthonous ARB, despite the relatively high detectable levels of human fecal markers. In contrast, the high resistome risk scores observed in chicken fecal samples may result from the selective pressure exerted by intensive antibiotic use, leading to the enrichment of ARBs, despite the relatively low detectable levels of human fecal markers. These findings suggest that while transient, anthropogenic inputs contribute to resistome risk scores, they do not fully explain the variability in resistome risk scores across different environments.

Other studies have proposed ranking the risks associated with individual ARGs in the environment [13, 49]. The MC2 framework is distinct in that it integrates the entire set of ARGs within an environmental sample, generating a sample-representative risk score. Using all ARGs detected in our dataset, we applied a Random Forest regression approach to identify ARGs that substantially contribute to ERR and HHRR in specific environments. We noted that *tetM* substantially contributed to HHRR in all environments (Fig. 6B). A previous study identified *tetM* as one of the most common ARG subtypes across six major habitats, including humans, farms, wastewater treatment plants, water, soil and air [50]. When comparing our list of ARGs that substantially contribute to the resistome risk scores with the ranked ARGs reported by Zhang et al., [13], we observed substantial overlap: 54.5% for ARGs with the ERR list and 76.9% for HHRR (Supplementary Table S8 and S9). Extending this approach, we also identified specific ARG-MGE combinations that substantially contributed to the risk scores. For HHRR, *tetM* co-occurring with an IGE, plasmid, or phage was a substantial contributor in 9/12 environments (Supplementary Table S12). For ERR, *bacA* co-occurring with either a phage or plasmid was a key contributor in 10/12 environments (Supplementary Table S11). A previous study reported the detection of *bacA* in all human fecal samples from 11 countries, suggesting that the gene may be ubiquitous in human populations [51]. Additionally, *bacA* was found to be among the top 10 most abundant ARGs in fecal metagenomes from Chinese, Danish, and Spanish populations [52]. Lastly, the *bacA* gene has also been found to be involved in virulence and biofilm formation in *Pseudomonas aeruginosa*, a member of the ESKAPEE pathogens, further highlighting its clinical relevance [53]. Given that bacitracin is a widely-used over the counter antimicrobial used to treat minor wounds, this presents concerns that this practice could be contributing broadly to the spread of AMR.

Among our list of ARG-MGE combinations that are substantial contributors to risk scores, we found plasmids and phages to be the common MGEs detected. Although it is challenging to definitively determine whether these MGEs are responsible for the dissemination of ARGs across environments in our dataset, phage-associated ARGs have been reported in previous studies across various environments, including human, freshwater, poultry, cattle, pig, and soil samples [5–56]. While the global nature of the dataset makes it difficult to determine the directionality of antibiotic resistance transfer between environments, the key ARGs and ARG-MGE combinations identified in this study may potentially serve as valuable targets for monitoring and mitigation efforts.

Finally, we also assessed the ARG abundance across the resistome profiles between environments. Chicken feces showed the highest ARG abundance, followed by hospital sewage, wastewater influent, and pig feces. In contrast, surface water, soil, activated sludge and sediment showed the lowest levels of ARG abundance. Similar trends have been reported in previous studies. Zhang et al. [13] and Yin et al. [58] also found the highest ARG abundances in chicken feces and the lowest in natural environments such as soil and sediment. While these studies reported pig feces as having the second highest ARG abundance, followed by human fecal samples, our findings identified hospital sewage as the second highest abundant environment. The high levels in hospital sewage are likely driven by the selective pressures exerted by the frequent use of antibiotics in clinical settings [59–60]. In poultry farming, the high ARG presence in chicken feces likely reflects widespread antibiotic use for both disease prevention and growth promotion, which have been linked to the emergence of resistant bacteria in their surrounding environments [61–62]. Among the ARGs identified, ARGs that confer resistance to tetracycline were particularly prevalent in farm animal fecal samples (chicken feces, pig feces, cow manure), a trend that is consistent with the widespread use of tetracyclines in agriculture [63–64]. From these sample types, cow manure showed the lowest abundance of tetracycline resistance, followed by chicken feces and then pig feces. Although publicly-accessible tetracycline usage data in livestock is limited, this observation is consistent with broader patterns of estimated antimicrobial usage in poultry, cattle and swine farming globally [65].

Given the inherent limitations of short-read Illumina data on the characterization of mobilized ARGs [66], the incorporation of long-read sequencing data into the MC2 framework could provide even more robust observations of resistome risk in complex environments. By combining the high accuracy of short-read data with the increased length of the long-reads, hybrid assembly has the potential to improve the detection and genomic contextualization of ARGs [67]. Additionally, development in bioinformatics tools for strain-resolved metagenomics can increase the specificity of host detection [68]. Improvement in Hi-C sequencing technology can increase the reliability of linking ARGs to their MGEs and host genomes [69]. Finally, the recovery of high-quality metagenome-assembled genomes for binning draft genomes of microbial populations, including those carrying ARGs. Collectively, these improvements and inclusion can improve the accuracy and ecological interpretation of the MC2 risk scores.

### Conclusions and Suggested Application

MC2 provides comparable metrics to users in the form of ERR and HHRR scores, which can be used to identify hot spots for AMR dissemination within and between environments of interest. Our findings suggest that the MC2 framework can effectively disentangle complex relationships between ARG abundance, composition, and environmental context, offering deeper insights into the underlying drivers that impact resistome risk. While this framework proved to be robust across a wide range of environments, we observed that in low-coverage, high-diversity samples, such as soil and sediment, the detection of ARG and their co-occurrences may be limited by technical constraints. While anthropogenic impact was positively correlated with increased resistome risk scores, it did not fully explain the variability observed across all environments. The improved understanding of technical, biological, and ecological factors underlying resistome risk scores across diverse environments provided by this study can help to tailor MC2 application towards appropriate use depending on the specific aims or goals of the surveillance or research effort. To improve the interpretability of the resistome risk scores, we recommend that users:

- Assess sequencing depth and assembly quality before drawing conclusions about ARG mobility.
- Supplement the MC2 resistome risk scores with contextual metadata such as anthropogenic inputs and sample source.
- Account for ecological factors, such as taxonomic diversity, which may influence ARG invasion and proliferation in an environment.

We further recommend the application of MC2 for:

- Relative comparisons across environments to identify general hotspots of interest for AMR dissemination to prioritize mitigation efforts.
- Focused comparisons across representative and comparable samples corresponding to specific environments of interest to inform targeted mitigation efforts.
- Integration into One Health surveillance frameworks to monitor AMR at the interface of human, animal and environmental health.

## Supplementary Material

Supplementary Files

This is a list of supplementary files associated with this preprint. Click to download.

• Additionalfile1.docx

• Additionalfile2.xlsx

## Data Availability

The datasets used in this study are downloaded from NCBI and their information has been shared through Supplementary Table S2.
